# Endometriosis in para-aortic lymph nodes without any pelvic endometriosis: the first case report, sequencing analysis and literature review

**DOI:** 10.1186/s12905-025-03964-0

**Published:** 2025-09-26

**Authors:** Yu Chen, Chunyan Hao, Qing Zhang, Qiuli Teng, Xiaoming Zhang, Guowei Tao, Lijie Wang

**Affiliations:** 1 Department of Obstetrics and Gynecology, Qilu Hospital of Shandong University, Ji’nan, Shandong 250012 P.R. China; 2 Gynecologic Oncology Key Laboratory of Shandong Province, Qilu Hospital of Shandong University, Jinan, 250012 P.R. China; 3 Department of Pathology, Qilu Hospital of Shandong University, Ji’nan, Shandong 250012 P.R. China; 4 Department of Radiology, Qilu Hospital of Shandong University, Ji’nan, Shandong 250012 P.R. China; 5 Department of Ultrasound, Qilu Hospital of Shandong University, Ji’nan, Shandong 250012 P.R. China

**Keywords:** Cystic adenomyosis, Endometriosis, Para-aortic lymph nodes, RNA sequencing, Whole-exome sequencing

## Abstract

This article introduces a novel case report of a patient exhibiting endometriosis within the para-aortic lymph nodes, concomitant with cystic adenomyosis and notably lacking pelvic endometriosis, alongside the sequencing analysis of her lesions. By synthesizing these findings with prior instances of lymph node endometriosis, the study seeks to offer innovative insights and identify potential targets for the diagnosis and treatment of this condition. A 36-year-old patient presented with escalating dysmenorrhea and a swiftly expanding abdominal mass. Imaging revealed the presence of a para-aortic mass, alongside a sizable cystic solid lesion in the pelvic abdomen featuring numerous internal grid-like patterns. The surgical removal of the uterus and enlarged para-aortic lymph nodes was performed via laparotomy, resulting in the pathological diagnosis of endometriosis in the para-aortic lymph nodes and cystic adenomyosis. Furthermore, sequencing analysis of lesions indicated that the lesions exhibited up-regulation of genes and pathways associated with cell activation, adhesion, inflammation, and immunity. Our study supports Halban’s “Benign Metastasis” theory in endometriosis, noting that lymph node spread often shows imaging changes and raised CA125 levels, necessitating distinction from malignant tumors for proper treatment. We also suggest a link between FOXA2 and SRRM2 genes and this endometriosis form, recommending further research on gene mutations and molecular pathways for better diagnosis and understanding.

## Introduction

Endometriosis, although considered a benign disease, exhibits certain characteristics commonly associated with malignancy, thereby posing significant threats to women’s quality of life and reproductive function. Primarily affecting pelvic tissues, endometriosis also manifests outside the pelvis in approximately 12% of cases, posting a clinical challenge due to its rarity and atypical symptoms [1]. The pathogenesis of endometriosis remains elusive, but Halban’s “Benign Metastasis” theory proposes endometriotic cells can spread to lymph nodes via lymphatic vessels (lymph node endometriosis, ICD: N80.8). Most studies focus on deep infiltration and intestinal endometriosis with local lymph node involvement. Research has shown that a positive lymph node with lesions extending beyond 1.75 cm or with 80% circumferential intestinal involvement may indicate a more aggressive form of the disease [2]. The occurrence of endometriosis in para-aortic lymph nodes is uncommon, with only four cases reported in PubMed [2–5], all of which were found to coexist with other forms of pelvic endometriosis (Table 1). The systematic search strategy employed was as follows: (“Endometriosis“[Mesh] OR “endometri*“[tiab] OR “endometrial implant*“[tiab]) AND (“Lymph Nodes“[Mesh] OR “lymph node*“[tiab] OR “lymphadenopathy“[tiab]) AND (“Aorta, Abdominal“[Mesh] OR “para-aortic“[tiab] OR “paraaortic“[tiab] OR “retroperitoneal“[tiab]). Aforementioned findings support the theory that endometriosis contributes to lymphatic metastasis. In contrast, our report describes a rare case of isolated endometriosis in the para-aortic lymph nodes, complicated by severe cystic adenomyosis, without pelvic endometriosis, indicating a unique form of the condition. In this context, “endometriosis” specifically means “classic endometriosis”, characterized by endometrial tissue (glands and stroma) outside the uterine cavity, unlike “adenomyosis”. While both are “endometriotic disorders”, they differ in location, symptoms, and underlying mechanisms. This indicates that ectopic endometrium in retroperitoneum can occur independently, reinforcing lymphatic metastasis as a mechanism. Additionally, our study is the first to provide sequencing data, identifying FOXA2 and SRRM2 genes as associated with this form of endometriosis.

**Table 1:** Reported cases of endometriosis in para-aortic lymph nodes

References	Publication year	Age, y	History	Symptoms	Pathology	Size of node
Beavis et al. [2]	2011	25	Bilateral ovarian endometriosis cysts removed	Placenta praevia; subchorionic hemorrhage; preterm abortion	Para-aortic endometriosis; pelvic endometriosis; ovarian endometriotic cysts	2.4 cm
Escobar et al. [3]	2013	23	Not mentioned	Pelvic pain	Para-aortic endometriosis; ovarian endometriotic cysts	Not mentioned
Christable et al. [4]	2021	26	Primary infertility; recurrence of ovarian endometriosis cysts after excision	Abdominal pain; vaginal bleeding	Para-aortic endometriosis; DIE; ovarian endometriotic cysts	3 cm
Li et al. [5]	2022	52	Hysterectomy (due to adenomyosis)	Progressive worsening of low back pain	Para-aortic endometriosis; ovarian endometriotic cysts	8 × 4 × 3cm^3^
Present case	2025	36	Adenomyosis(taking mifepristone for three years)	Significantly enlarged abdominal mass	Para-aortic endometriosis; adenomyosis	5 × 4 × 3cm^3^

## Case report

**Fig. 1:**
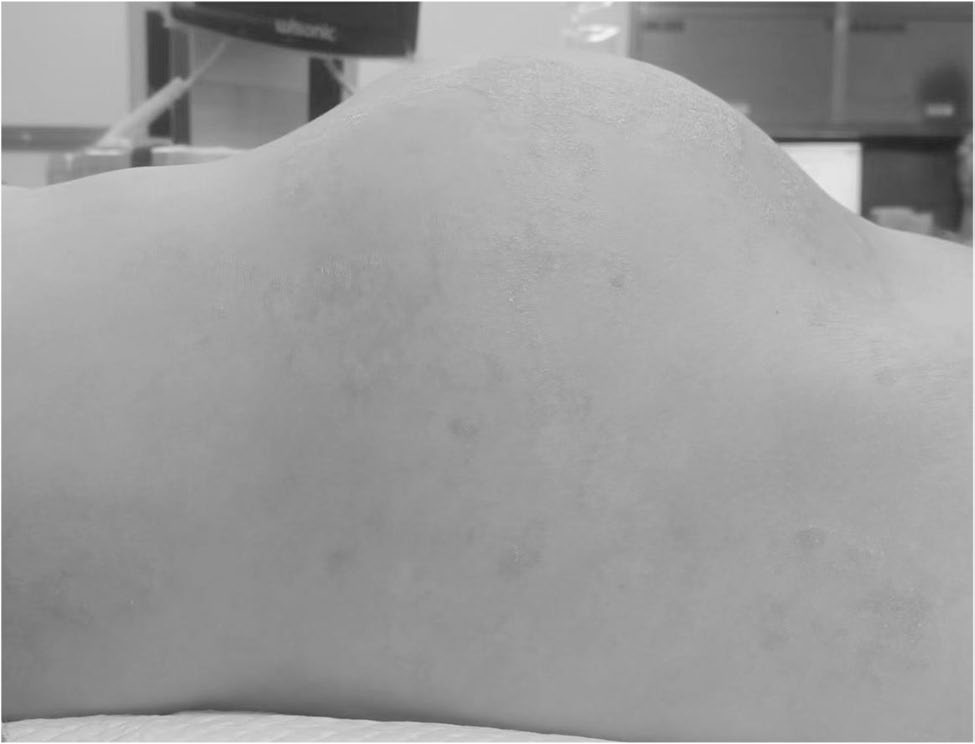
Patient’s abdomen in a lying position

**Fig. 2:**
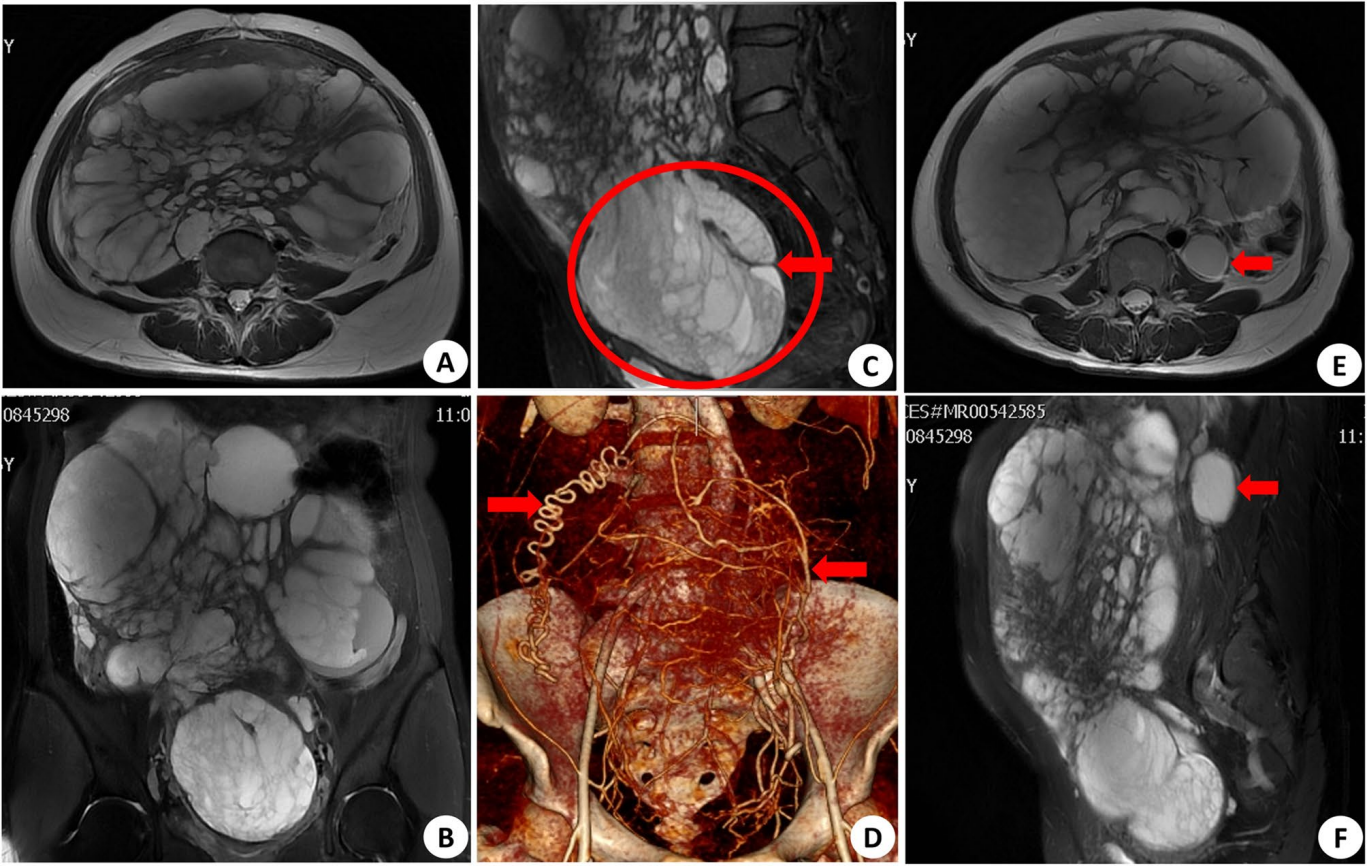
Imaging manifestations of the patient. Axial (**A**), coronal (**B**) and sagittal (C) nonenhanced MRI of the pelvis demonstrates enlarged uterus, the circle and the arrow show distorted cervix and an open external cervical opening and vaginal vault. CT three-dimensional reconstruction (**D**) showed abundant blood supply of the uterus (arrow). Axial (**E**) and sagittal (**F**) MRI of the lymph nodes in the left retroperitoneum of aorta (arrow)

A 36-year-old patient presented with escalating dysmenorrhea for 9 years and a swiftly expanding abdominal mass for 3 months. Throughout the progression of the disease, the patient demonstrated increasingly severe dysmenorrhea of extended duration, accompanied by a progressively enlarging mass in the lower abdomen. It is noteworthy that the patient self-administered mifepristone (Patient could not recall the dose) for a period of three years to manage intolerable and persistent lower abdominal pain. Symptom relief during medication, nonetheless, following the cessation of the medication, there was a notable recurrence of symptoms and an accelerated progression of the disease. The patient’s abdomen exhibited significant enlargement in the upright position, comparable to the size of a 5-month pregnancy (Fig. 1). Enhanced CT and MRI scans (Fig. 2) revealed the presence of a para-aortic mass measuring 4.2 cm, alongside a sizable cystic solid lesion in the pelvic abdomen featuring numerous internal grid-like patterns and a rich blood supply measuring approximately 23.5 × 12.4 × 27.0 cm^3^. Elevated levels of CA125, CA199 and prolactin were detected, measuring 1115 U/ml, 464 U/ml and 904.3µIU/ml, respectively. The patient’s unique imaging, high tumor markers, and swift disease progression necessitated careful preoperative differentiation from uterine malignancies.

**Fig. 3:**
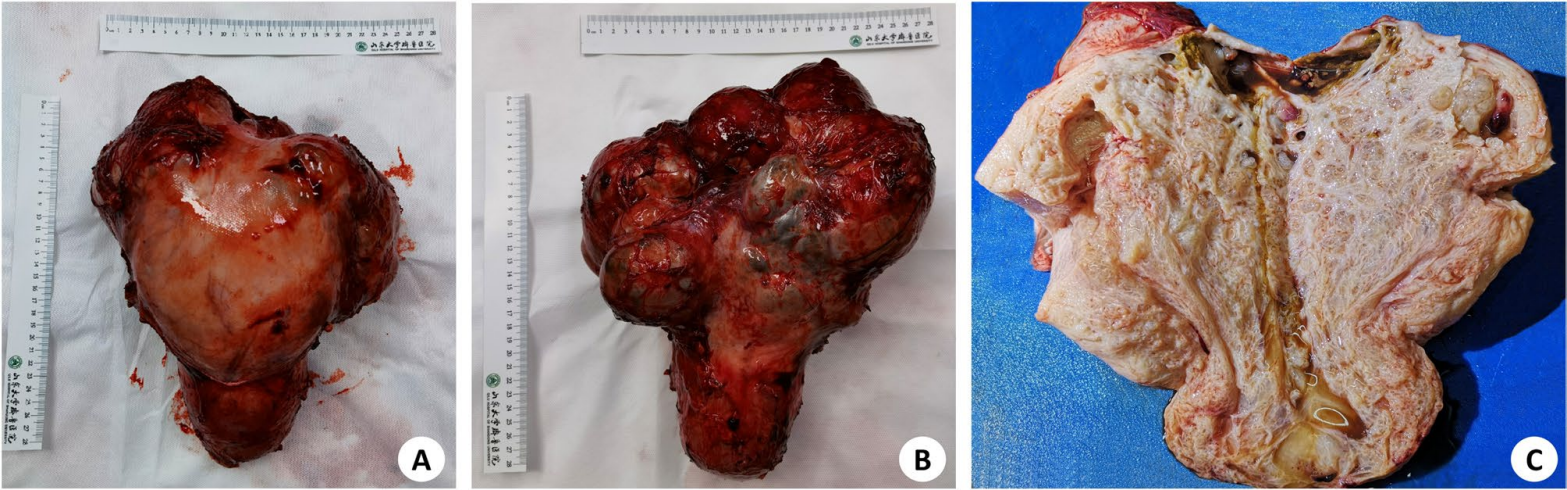
Anatomical view of the uterus. **A** Front view. **B** Back view. **C** Cutaway view

The patient underwent an exploratory laparotomy, with the explicit request of not preserving reproductive function. Intraoperatively, the uterus was observed to be extensively distorted measuring 30 × 20 × 15 cm^3^ (Fig. 3), identifing multiple cystic massesin the posterior wall of the uterus, with the largest one measuring 6 cm in diameter. A circular mass, exhibiting clear boundaries and measuring approximately 5 × 4 × 3cm^3^, was identified in close proximity to the aorta. No evident abnormalities were detected in the other abdominopelvic organs and peritoneum. Subsequently, the patient underwent surgical removal of the uterus, bilateral fallopian tubes, omentum, para-aortic lymph nodes, and para-aortic masses.

**Fig. 4:**
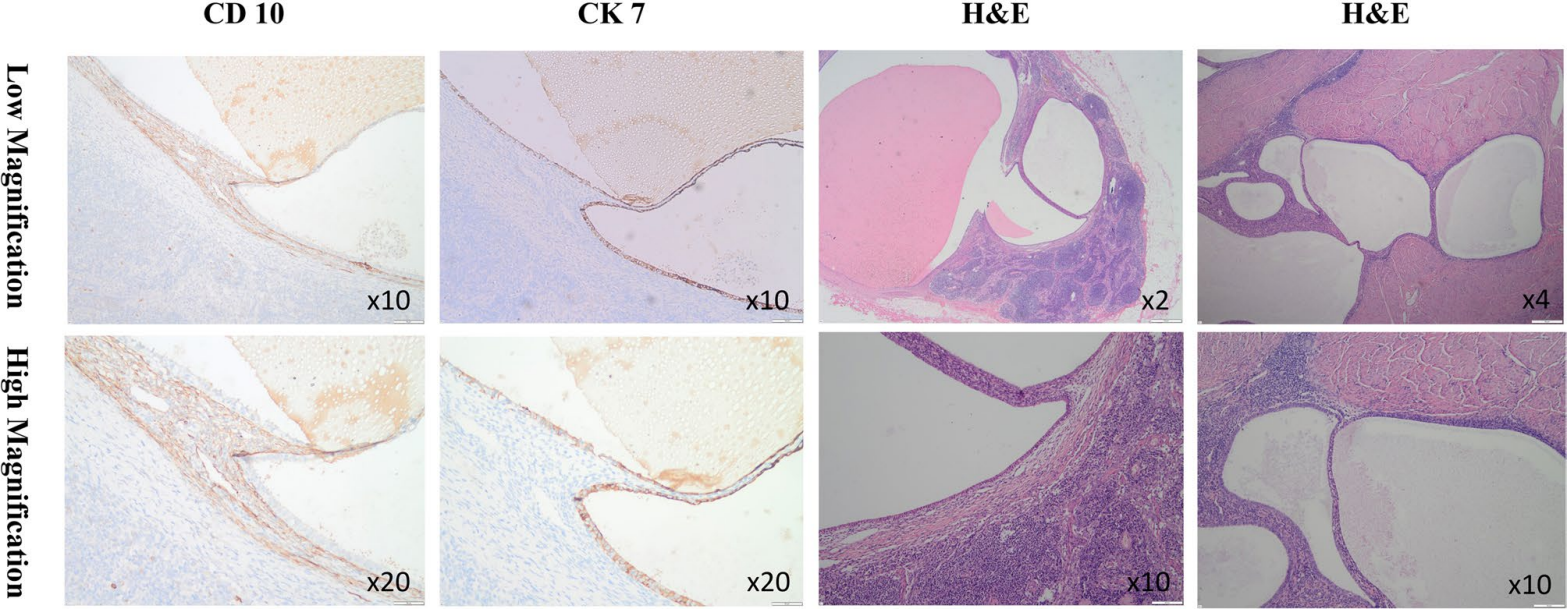
Histologic appearance of endometriotic implant in para-aortic lymph nodes. H&E = hematoxylin and eosin

The final histologic pathology confirmed the uterine adenomyosis, endometriosis, and endometriotic cyst in the para-aortic lymph nodes. Immunohistochemical staining demonstrated positive expression of CK7, PAX-8, WT-1, and CD10 (Fig. 4).

For two years post-surgery, regular follow-ups with pelvic ultrasounds, abdominopelvic CT scans, tumor marker tests, and routine gynecological exams were conducted, revealing no abnormalities.

**Fig. 5:**
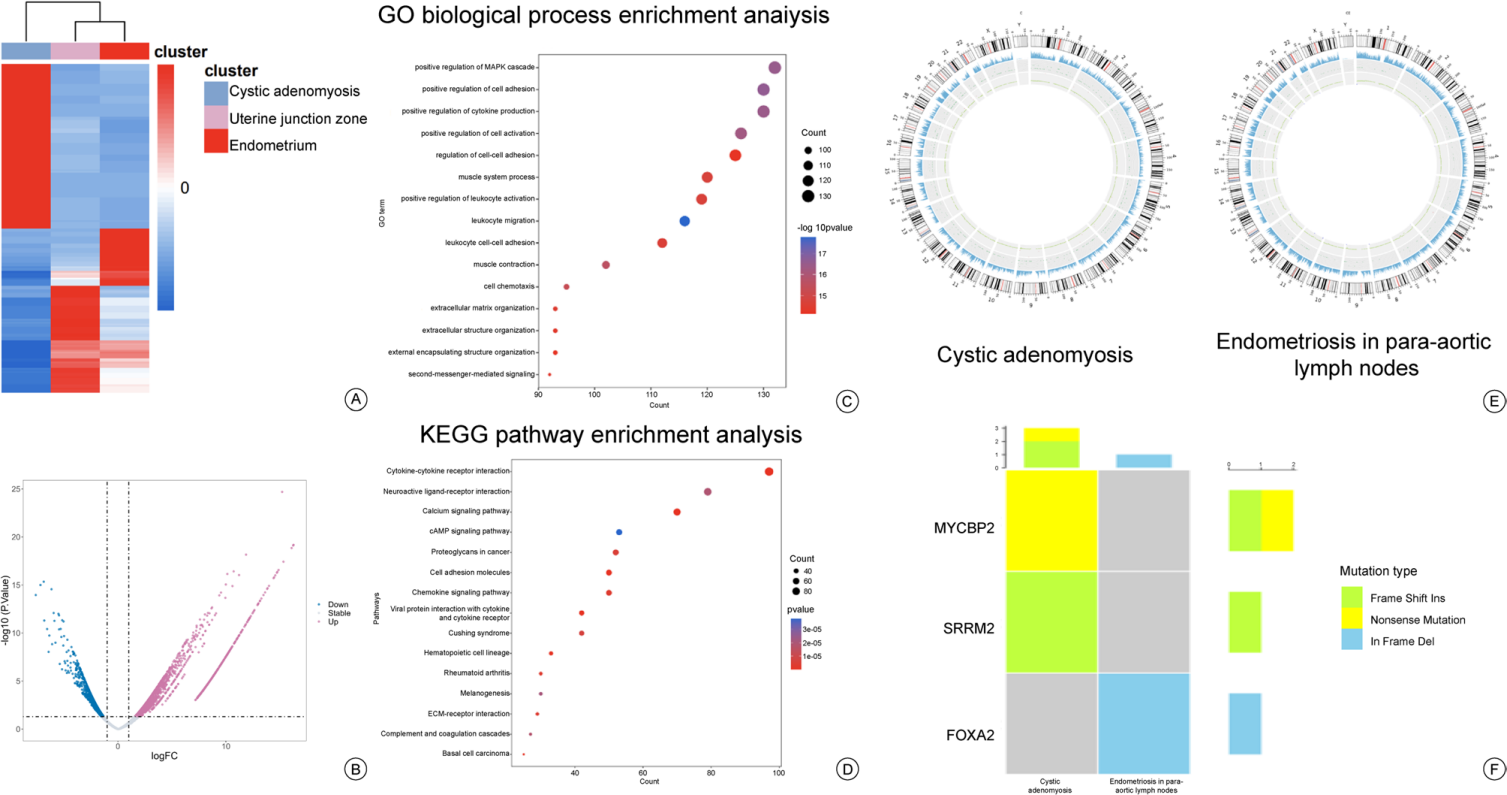
Cystic adenomyosis lesions were compared with endometrium and uterine junction zone (from the patient’s own tissue) by RNA-seq, both cystic adenomyosis and endometriosis in para-aortic lymph nodes were compared with blood by WES. **A** Hierarchical clustering heatmap based on the differentially expressed genes obtained through RNA-seq shows that the cystic adenomyosis lesions are significantly different from endometrium and uterine junction zone. **B** Volcano plot of differentially expressed genes between cystic adenomyosis lesions and endometrium as well as uterine junction zone. Each point represents a detected gene. The number of the upregulated genes and downregulated genes are 1927 and 1275 respectively. |log2 Fold Change|>1 and an adjusted *p* value <0.05 were considered significant. **C** and **D** are the GO and KEGG enrichment analysis results of the cystic adenomyosis lesions compared with endometrium and uterine junction zone, respectively. **E** and **F** are the genomic variation circles and driver gene screening results of the cystic adenomyosis and endometriosis in para-aortic lymph nodes compared with blood, respectively

We conducted sequencing analysis on the lesions of the patient (Fig. 5). RNA-seq of cystic adenomyosis lesions indicate that the differentially expressed genes primarily contribute to the upregulation of cell activation, cytokine production, cell-cell adhesion, and leukocyte activation. The WES analysis indicated that FOXA2 and SRRM2 were respectively candidate driver genes for endometriosis in para-aortic lymph nodes and cystic adenomyosis requiring validation in larger cohorts and functional studies.

## Discussion

The mechanism of lymphatic metastasis in endometriosis remains unclear. Halban’s theory of “Benign Metastasis” proposes that benign endometrial stroma and glands can disseminate through lymphatic and blood vessels. Previous research has demonstrated that individuals diagnosed with endometriosis exhibit heightened lymphangiogenic potential and an increased density of lymphatic vessels in the eutopic endometrium, it can also facilitate the infiltration of endometrial tissue into the lymphatic circulation [6]. Our sequencing findings indicate an upregulation of genes and pathways associated with cell activation, adhesion, and inflammation in cystic adenomyosis lesions. Consequently, these molecular alterations may contribute to an augmented presence of blood vessels and lymphatic vessels in the lesion, thereby facilitating the metastasis, colonization, and growth of ectopic endometrial cells in distant lymph nodes. A research revealed the consistent presence of CD10 + endometrial stromal cells in obturator lymph nodes throughout the menstrual cycle, with their numbers peaking during menstruation [7]. Additionally, that the increased density of endometrial lymphatic microvessels may enhance the migration of endometrial cells to the draining lymph nodes.

Studies and reports on lymph node endometriosis are limited and its exact prevalence is unknown. In a prospective study [8], 2 of the 19 sentinel lymph nodes (11%) in different patients with endometriosis had endometrial cell sentinel lymph node involvement, which can be a source of recurrence of endometriosis if not removed. A observational study related to laparoscopic tubal patency [9] observed dye penetration of the uterine wall in 27 patients (26%) with adenomyosis and showed clear dye channels in the pelvic lymphatics. It was found that under the influence of adenomyosis, its ectopic endometrium has increased vascular and lymphatic vascular production [10]. Lymphatic dilation as a feature of adenomyosis allows the drainage of intrauterine fluid (dye or menstrual blood) from the uterine cavity to extrauterine organs via the lymphatic network at minimal intrauterine pressure. This provides a theoretical basis for the occurrence of severe cystic adenomyosis with distant lymph node metastasis in our case.

Contrary to previous studies, our report suggests ectopic endometrium in retroperitoneum can occur independently, which means endometrium is more aggressive in this patient. The “eutopic endothelial determinism” points out that ectopic endothelium causes disease through attachment, aggression, and angiogenesis (AAA) [11], which shares similar characters with malignancy. This pathogenic process is based on the different physiological characteristics and genetic differences of eutopic endothelium. Epidemiology has shown that ovarian endometriosis is closely associated with ovarian clear cell carcinoma and endometrioid carcinoma [12]. A gene expression analysis of endometriosis spreading to the sentinel pelvic lymph nodes showed that this process was accompanied by differential gene expression of many specific genes, such as upregulation of CD 44 variants, and loss of expression of epithelial markers [13]. A study on lung cancer found primary tumors with higher CD44 expression increased metastasis to regional lymph nodes, and an enrichment of CD44 + cells in metastatic lymph nodes compared to primary tumors [14]. Similarly, serum exosome CD44 concentration was positively correlated with lymph node tumor load and could be used as a potential therapeutic target and non-invasive biomarker for lymph node metastasis in gastric cancer [15]. Thus, upregulation of CD44 expression may also play a role in lymph node metastasis and its increased aggressiveness in the endometrium. Loss of ARID1A expression playing an important role in the malignant process of endometriosis [16], a study also found that partial loss of ARID1A expression was detected in ovarian endometriosis, DIE, and endometriosis in pelvic sentinel lymph nodes [17]. Our sequencing analysis suggested that FOXA2 and SRRM2 may respectively be candidate driver gene of endometrial metastasis to lymph nodes and myometrium. FOXA2 has a high mutation rate in aggressive variants of endometrial cancers (ECs). Research indicates that inactivating both FOXA2 and RTEN leads to lethal endometrial cancers, with FOXA2 suppressing carcinogenesis [18]. Downregulation of nuclear FOXA2 is linked to breast cancer metastasis via the AKT pathway [19]. SRRM2 is upregulated in colon adenocarcinoma, predicting poor prognosis [20], and its silencing inhibits angiogenesis in nasopharyngeal carcinoma through MYLK-mediated cGMP-PKG signaling pathway [21]. FOXA2 and SRRM2 are potential biomarkers for yolk sac tumors and Alzheimer’s disease [22, 23]. Mutations in these genes may relate to metastasis in intimal cells and other genes involved in endometriosis malignancy. Further research is needed to explore their clinical significance and potential as biomarkers for severe endometriosis and adenomyosis, which could lead to timely interventions to preserve fertility.

Endometriosis diagnosis typically relies on patient history, symptoms, and tests, but lymph node endometriosis is often mistaken for cancer due to a lack of reliable markers. Diagnosis depends on surgeon experience and imaging. Advancements in noninvasive diagnostics and a better understanding of adenomyosis have revealed its strong link to adolescence, often appearing in early puberty and worsening over time. Early diagnosis is crucial for effective management [24]. Therefore, clinicians should maintain sufficient awareness and vigilance toward symptoms such as dysmenorrhea to facilitate early detection and treatment of the disease. Adenomyosis often occurs alongside endometriosis, which also appears early, so screening for both, especially deep posterior endometriosis lesions, is recommended. In instances of intricate clinical scenarios, such as substantial adenomyosis lesions or the swift enlargement of lesions over a brief duration, a systematic imaging assessment of the pelvic and para-aortic lymph nodes is justified. Recent studies have shown that functional vascular endothelial growth factor (VEGF)-C promotes lymphangiogenesis and immune cell infiltration in endometriosis via extracellular vesicle (EVs) transport, which is a reliable noninvasive clinical diagnostic method through evaluating EV-transmitted VEGF-C in patients’ serum [25]. This can help assess the risk of lymphatic involvement and guide treatment. Postoperative pathology confirms the diagnosis, and any lymph node endometriosis found during surgery should be removed to minimize recurrence. However, the best treatment protocol requires confirmation through large randomized trials.

Due to the rarity of this case, sequencing was limited to one patient, preventing large-scale analysis. The study also lacks experimental validation. Future clinical efforts will focus on collecting samples from similar cases to enable broader analysis and better evidence. Large-scale studies should clarify lymph node involvement in different endometriosis types. Fundamental research is needed to establish lymphatic metastasis theory and identify biomarkers for severe endometriosis and adenomyosis. These efforts are crucial for timely, standardized diagnosis and treatment to protect fertility.

## Data Availability

No datasets were generated or analysed during the current study.

## Abbreviations

CA125: Carbohydrate Antigen 125
CA199: Carbohydrate Antigen 199
CT: Computed Tomography
MRI: Magnetic Resonance Imaging
WES: Whole-exome sequencing
ECs: Endometrial cancers
EVs: Extracellular vesicles
